# An examination of internet and land-based gambling among adolescents in three Canadian provinces: results from the youth gambling survey (YGS)

**DOI:** 10.1186/s12889-016-2933-0

**Published:** 2016-03-18

**Authors:** Tara Elton-Marshall, Scott T. Leatherdale, Nigel E. Turner

**Affiliations:** Social and Epidemiological Research, Centre for Addiction and Mental Health, 100 Collip Circle, Suite 200, London, N6G 4X8 Ontario Canada; Dalla Lana School of Public Health, University of Toronto, Toronto, Ontario Canada; Department of Epidemiology and Biostatistics, Western University, London, Ontario Canada; School of Public Health and Health Systems, University of Waterloo, Waterloo, Ontario Canada

**Keywords:** Adolescent, Gambling, Online, Prevalence

## Abstract

**Background:**

With the rapid proliferation of new gambling technology and online gambling opportunities, there is a concern that online gambling could have a significant impact on public health, particularly for adolescents. The aim of this study is to examine online and land-based gambling behaviour among adolescents in 3 Canadian provinces (Ontario, Newfoundland and Labrador, Saskatchewan) prior to the implementation of legalized online gambling.

**Methods:**

Data are from 10,035 students in grades 9 to 12 who responded to the 2012–2013 Youth Gambling Survey (YGS) supplement, a questionnaire administered as part of the Canadian Youth Smoking Survey (YSS, 2012) in 3 provinces: Newfoundland and Labrador (*n* = 2,588), Ontario (*n* = 3,892), and Saskatchewan (*n* = 3,555).

**Results:**

Overall, 41.6 % of adolescents (35.9 % of females and 47.4 % of males) had gambled in the past 3 months. 9.4 % of adolescents had gambled online in the past 3 months alone (3.7 % of females and 15.3 % of males). The most popular form of online gambling was online sports betting. Adolescents also engaged in online simulated gambling including internet poker (9.1 %) and simulated gambling on Facebook (9.0 %). Few adolescents participated in online gambling exclusively and online gamblers were more likely than land-based gamblers to engage in multiple forms of gambling. A higher proportion of adolescent online gamblers scored “high” or “low to moderate” in problem gambling severity compared to land-based only gamblers.

**Conclusions:**

Despite restrictions on online gambling at the time of the study, adolescents were engaging in online gambling at a significantly higher rate than has been previously found. Adolescents were also using technology such as video games to gamble and free online gambling simulations.

## Background

Problem gambling is associated with numerous negative health and mental health outcomes including co-morbid substance use [1], depression, anxiety, and suicide [2], and poor overall health [3]. Gambling has changed dramatically in a short period of time [4]. With the introduction of new technological advances in gambling such as online gambling, opportunities to gamble have become more abundant. At the same time, governments in numerous jurisdictions have begun to expand gambling opportunities to include online gambling platforms as a way to increase tax revenues [5]. Of particular concern for public health is the impact of the expansion of legalized gambling and online gambling on the current generation of youth [6, 7].

A recent review of the evidence demonstrated that few studies have examined internet gambling among youth [8]. The research that has been conducted suggests that prevalence rates among youth are low but are predicted to increase as more opportunities become available [8]. It is estimated that between 2003 and 2014 the online gaming market grew from $7.4 to $39.5 billion USD [9]. Additionally, youth are more exposed to opportunities to practice gambling through free demo versions of games, integration into social networking sites like Facebook, and in video games [8]. A recent study conducted in Australia suggests that a significant proportion of youth were engaging in simulated gambling [10]. This is cause for concern because adolescents who engage in simulated gambling are potentially at greater risk for problem gambling [10].

There is a range of variability in government run online gambling in Canada. While some provinces do not currently plan to offer government run online gambling (Saskatchewan), others offer lottery tickets or iBingo but no other forms of online gambling (the Atlantic Provinces of New Brunswick, Nova Scotia, Prince Edward Island, and Newfoundland and Labrador) and others have already legalized online gambling (British Columbia, Quebec, Manitoba, Ontario). Monitoring gambling in Ontario is particularly important as the government introduced an online gambling website in January 2015 and modernization plans have also included the introduction of more casinos throughout the province [5].

Most recent estimates suggest that gambling prevalence among youth in Canada is high. Approximately 80 % of adolescents in Canada have gambled at least once in their lifetime [2, 11]. National prevalence estimates of moderate-risk or problem gambling are 2.2 %, with males being more likely to report problems than females [12]. The majority of surveys conducted in Canada examining adolescent gambling have used convenience samples of adolescents typically located close to major city centers [13]. Current estimates at the provincial level are lacking with extreme variability in what data has been collected to measure adolescent gambling prevalence [13]. The current study then will examine the prevalence of youth gambling among adolescents in 3 provinces: Ontario, Newfoundland and Labrador, and Saskatchewan. Data for this study were initially collected as part of a larger host study designed to gather baseline data prior to the legalization of online gambling in Ontario (compared to two control provinces with no legislative changes).

Most recent prevalence data from Ontario found that in 2013, 35 % of youth reported gambling at least once in the past 12 months. Approximately 3.1 % of youth had participated in online gambling over the previous 12 months. Most recent estimates from Saskatchewan suggest that in 2005, 81 % of youth aged 15 to 18 participated in gambling [14]. Very little information is known about the prevalence of youth gambling in Newfoundland and Labrador at any time point. A 1998 study in all of the Atlantic provinces demonstrated that 70.3 % of youth had gambled in the past 12 months [15]. The prevalence of online gambling is unknown in these provinces.

There was a decline in most youth gambling activities in Ontario since 2003 except for online gambling which has remained stable [16]. Playing card games (10.7 %) and betting in sports pools (10.2 %) were the most prevalent land-based gambling activities in Ontario, whereas casino gambling (prohibited to those under 19) was the least prevalent (>1 %) in both Ontario and Saskatchewan. Males were significantly more likely than females to gamble [14, 16] and to report multi-gambling activity [16]. Older studies in Saskatchewan and Newfoundland and Labrador suggest that scratch tickets, playing cards for money, playing the lottery and games of skill were among the most prevalent gambling activities, but this may reflect the fact that the research was conducted much earlier and gambling opportunities have changed since these surveys were conducted [14, 15]. Further information about the types of online gambling activities is not known.

The current study advances the literature in several ways. With the increased proliferation of online gambling websites timely data is needed to determine the prevalence of online gambling among adolescents and to identify the ways that adolescents are engaging in online gambling, particularly as the gambling technology changes. This information can be used to inform improved responsible gambling practices. Few studies have been conducted in Newfoundland and Labrador and Saskatchewan and those that have been done were several years ago. There is a need for more research that uses similar methodology to compare regional differences [17]. Furthermore, whereas previous research has used measures of gambling in the past 12 months to determine current gambling behaviour this study uses gambling in the past 3 months. The 3 month time frame is used as part of the Canadian Adolescent Gambling Inventory (CAGI), the first measure of adolescent gambling designed and tested specifically with adolescents [18]. This is the first study to use the CAGI to assess gambling prevalence among youth in these 3 provinces.

The study has 3 objectives: (1) to understand how adolescents are engaging in gambling online and offline; (2) to determine the prevalence of online gambling and free simulated gambling; and (3) to compare problem gambling prevalence rates between online and land-based gamblers.

## Methods

### Design

Data are from 10,035 students in grades 9 to 12 (aged 13–19) who responded to the 2012–2013 Youth Gambling Survey (YGS) supplement. The YGS was implemented as a supplemental questionnaire to the Canadian Youth Smoking Survey (YSS, 2012) in 3 provinces: Newfoundland and Labrador (*n* = 2,588), Ontario (*n* = 3,892), and Saskatchewan (*n* = 3,555). Participating YSS boards, schools, and students had the option to participate in the YGS. 78 schools were approached; 39 participated and 39 refused. Only 3 school boards (2 Ontario, 1 Saskatchewan) and 3 schools (2 Newfoundland and Labrador, 1 Ontario) chose to participate in the YSS survey but not the YGS. 92 % of students who completed the YSS also completed the YGS survey. The YSS is a representative school-based survey of youth in 9 provinces in Canada. Schools that did not contain students in Grades 9 to 12 or federally funded schools such as: schools on First Nations Reserves, schools for special needs children, and schools with fewer than 20 students in the eligible grades were excluded. Eligible schools were identified and sampled by health region smoking rates (consistent with YSS protocols). Detailed information on the 2012-13 YSS design and methods, as well as the YGS supplement are available online (https://uwaterloo.ca/canadian-student-tobacco-alcohol-drugs-survey) [19]. The survey design and sample weights for the YGS as part of the YSS allow us to produce population-based weighted sample estimates within this manuscript.

### Ethics, consent and permissions

The University of Waterloo Office of Research Ethics and appropriate School Board and Public Health Ethics committees approved all procedures for the YSS and YGS supplement. For schools that required active permission protocols, parents were sent information letters about the project and asked to return permission forms. For schools that required active information but passive permission, parents were provided with information letters about the study and were given a toll-free number to call if they did not want their child to participate. Students in all schools had permission to decline participation on the day of the data collection. Data was collected by the study authors and therefore no permission was required to publish these findings.

### Measures

The YGS measures on gambling activities in the past 3 months are adapted from the Canadian Adolescent Gambling Inventory (CAGI) with consideration given to emerging priorities (e.g. specific types of internet gambling) [20]. We did not incorporate measures of time spent gambling for each of the activities (which was beyond the scope of the study and not a priority for questionnaire time/space).

#### Types of gambling

Respondents were asked to report how often in the last 3 months they bet or gambled money or something of value in 16 different gambling activities (see Table 1). Response options were “not in the past 3 months,” “about once per month,” “2–3 times per month,” “about once per week,” “2–6 times per week” and “daily”. Gambling frequency was coded as “at least monthly but less than weekly” if respondents gambled “about once per month” or “2–3 times per month”; and “at least weekly” if respondents gambled “about once per week,” “2–6 times per week,” or “daily”. The overall prevalence was based on any participation (indicated “about once per month” or more frequent).

**Table 1:** Prevalence of participation in gambling behaviours by gender, Grades 9 to 12 students in the Youth Gambling Survey (YGS) Saskatchewan, Ontario, and Newfoundland and Labrador (Canada, 2012-2013)

	*Gambling Participation (past 3 months)*			
	Male	Female	Total	
	(*n* = 4,937)	(*n* = 5,098)	(*n* = 10,035)	
Gambling Behaviours Played *for Money*	%^a^	%^a^	%^a^	Chi-Square
Internet Gambling				
Internet Poker	6.2	1.1^b^	3.6	*X* ^*2*^ = 34.1(1), *p* < 0.001
Sports Pools (e.g., Hockey, Baseball) Online	12.1	2.7	7.3	*X* ^*2*^ = 45.3(1), *p* < 0.001
Slot Machines Online	3.3	1.7^b^	2.5	*X* ^*2*^ = 4.7(1), *p* = 0.03
Overall Participation in Internet Gambling	15.3	3.7	9.4	*X* ^*2*^ = 34.5(1) *p* < 0.001
Land Based Gambling				
Sports Pools (e.g., Hockey, Baseball) Not Online	14.2	3.9	9.0	*X* ^*2*^ = 132.5(1), *p* < 0.001
Slot Machines Not Online	2.7^b^	NR	1.9^b^	*X* ^*2*^ = 5.70(1) *p* = 0.02
Arcade or Video Games	14.5	5.1	9.7	*X* ^*2*^ = 140.9(1), *p* < 0.001
Sport Select (e.g., Pro Line, Over/Under)	12.3^b^	2.2^b^	7.2	*X* ^*2*^ = 46.7(1), *p* < 0.001
Lottery Tickets (e.g., 6/49, Super 7)	10.8	8.5	9.6	*X* ^*2*^ = 5.5(1) *p* = 0.02
Instant-Win or Scratch Tickets	14.2	13.3	13.8	*X* ^*2*^ = 0.6(1) *p* = 0.44
Cards (e.g., Poker, Black Jack)	14.4	3.2^b^	8.7	*X* ^*2*^ = 68.7(1), *p* < 0.001
Board Games or Dice	9.5	2.2^b^	5.8	*X* ^*2*^ = 51.5(1), *p* < 0.001
Video Lottery Terminals	3.8	0.9^b^	2.4	*X* ^*2*^ = 20.0 (1), *p* < 0.001
Horse Races (at track or off-track)	3.8	2.1^b^	3.0	*X* ^*2*^ = 6.3(1), *p* = 0.01
Games of Skill (e.g., pool, darts, etc.)	16.6	8.4	12.4	*X* ^*2*^ = 172.2(1), *p* < 0.001
Dare or Challenge	24.5	19.8	22.1	*X* ^*2*^ = 29.2(1), *p* < 0.001
Bingo	6.5	7.0	6.8	*X* ^*2*^ = 1.06(1), *p* = 0.30
Internet Gambling Behaviours Played *for Fun (no money)*				
Internet Poker	14.6	3.8	9.1	*X* ^*2*^ = 81.8(1), *p* < 0.001
Internet Slots	6.6	3.3	4.9	*X* ^*2*^ = 33.6(1), *p* < 0.001
Gambling Games on Facebook	11.7	6.4^b^	9.0	*X* ^*2*^ = 14.0(1), *p* < 0.001

^a^Weighted population estimate

^b^Moderate sampling variability, interpret with caution

^NR^High sampling variability or low sample size, data are suppressed

#### Free simulated gambling

Respondents were asked whether they had participated in any online gambling activities for fun (no money) in the past 3 months. These activities were: (1) internet poker; (2) internet slots; and (3) gambling games on Facebook. Response options were consistent with measures of types of gambling.

#### Gambling participation

Respondents were identified as having participated in gambling if they reported gambling at least once in the past 3 months for one or more of the 16 gambling activities measured.

#### Online vs. land-based gamblers

Online gamblers were any respondents who indicated that they had gambled money or something of value in the past for any of 3 online gambling activities: (1) internet poker; (2) sports pools online; (3) slot machines online. Land-based gamblers were any respondents who had gambled money or something of value in the past 3 months but had not participated in any of the online gambling activities.

#### Gambling across multiple types of games

A measure of co-occurring gambling behaviour was calculated based on individual responses to the 16 gambling activities to determine if respondents who gambled online were more likely to participate in more than one type of gambling behaviour.

#### Problem gambling

Problem gambling was measured using the 9 item Gambling Problem Severity Subscale (GPSS) of the CAGI which is the only problem gambling measure developed specifically for adolescents [18]. Further details about the GPSS and its development can be found elsewhere [20].

### Analyses

Descriptive analyses of the types of gambling were examined by gender for the whole sample and by age for adolescents who had engaged in gambling. Descriptive cross tabulation analyses were used to examine the prevalence of engaging in online simulated gambling by gambling participation. Among adolescents who had gambled, we examined the prevalence of engaging in online, land based or both online and land based gambling. Gambling across multiple types of games was compared between online and land-based (those who never gambled online) platforms. Among online gamblers only we examined the types of games played online. Among adolescents who had gambled, cross tabulations were used to examine differences in the prevalence of problem gambling by gambling type (online vs. land-based only). In all analyses, survey weights were used to adjust for non-response between provinces and groups, thereby minimizing any bias in the analyses caused by differential response rates across regions or groups. Bootstrap weights were used for all significance tests so that the variances take account of the sample design. Significance was assessed using the first-order Rao-Scott chi-square test. For missing data, imputations were not performed; as such, the prevalence of each risk factor was based on the sample that had complete data for that particular indicator. This allowed us to preserve as much of the sample data as possible. Survey questions related to use of “free games” online had a higher proportion of missing data but sensitivity analyses demonstrated that including missing data did not significantly change the prevalence. For example, “internet slots for free” had the most missing data (*n* = 826 missing). With no missing data included the prevalence was 4.9 % with missing data included the prevalence was 4.5 %. The statistical package SAS 9.4 was used for all analyses.

## Results

The sample population was 49.3 % male and 50.7 % female. The mean age was 16.5 (SE = 0.1). The mean age for females was 16.4 (SE = 0.2) and 16.6 (SE = 0.1) for males. The mean age by province was 16.5 (SE = 0.1) in Ontario, 16.3 in Saskatchewan (SE = 0.2) and 16.3 in Newfoundland/Labrador (SE = 0.2). Overall, 41.6 % of youth in our sample were current gamblers (35.9 % of females and 47.4 % of males).

### Gambling behaviour by gender

Table 1 presents the weighted prevalence estimates of different gambling behaviours by gender. The overall prevalence of online gambling among all adolescents was 9.4 % (3.7 % of females and 15.3 % of males). The most common form of online gambling was online sports pools (7.3 %) and this pattern was consistent for both males (12.1 %) and females (2.7 %). The prevalence of online gambling did not differ by province (*p* = 0.56) (Ontario: 9.5 %; Newfoundland and Labrador: 9.0 %; Saskatchewan: 8.3 %). The most prevalent types of gambling behaviour for money or something of value were land-based: (1) participating in a dare or challenge (22.1 %); (2) instant-win or scratch tickets (13.8 %); and (3) games of skill (12.4 %). Males were more likely to participate in all forms of gambling except Bingo and instant-win or scratch tickets which were not significantly different by gender.

### Gambling participation by age

Table 2 presents the weighted prevalence of gambling participation by age. There was significant variability in internet poker participation by age with the highest proportion of adolescents being 17 years old. Similarly, the highest proportion of adolescents participating in sports pools (not online) were 16. The highest proportion of respondents buying lottery tickets or instant win/scratch tickets were 18 years of age or older.

**Table 2:** Prevalence of current participation in gambling behaviours by age, Grades 9 to 12 students in the Youth Gambling Survey (YGS) Saskatchewan, Ontario, and Newfoundland and Labrador (Canada, 2012-2013)

	*Current Participation (past 3 months)*				
	14 years or younger	15 years	16 years	17 years	18 years or older
	(*n* = 1,883)	(*n* = 2,641)	(*n* = 2,490)	(*n* = 2,140)	(*n* = 824)
Gambling Behaviours Played *for Money*	%^a^	%^a^	%^a^	%^a^	%^a^
Internet Gambling					
Internet Poker**	9.7^b^	24.5	19.9	33.4	12.5^b^
Sports Pools (e.g., Hockey, Baseball) Online	15.7	27.0	22.6	26.9	7.7
Slot Machines Online	17.3^b^	20.8^b^	17.8^b^	25.6^b^	18.5^b^
Land Based Gambling					
Sports Pools (e.g., Hockey, Baseball) Not Online*	13.3	23.4	28.5	26.5	8.2^b^
Slot Machines Not Online**	8.7^b^	15.9^b^	17.4^b^	33.4^b^	NR
Arcade or Video Games	16.9^b^	27.3	24.0	20.8	11.0^b^
Sport Select (e.g., Pro Line, Over/Under)	11.9	22.5	25.0	30.2	10.4^b^
Lottery Tickets (e.g., 6/49, Super 7)***	7.2	15.2	19.0	26.4	32.2
Instant-Win or Scratch Tickets***	12.8	18.6	19.9^b^	24.3	24.5^b^
Cards (e.g., Poker, Black Jack)	12.4^b^	20.2	21.7	33.4	12.2
Board Games or Dice	10.6^b^	22.4	26.9	30.2	9.9
Video Lottery Terminals	NR	21.5^b^	20.6	31.9^b^	NR
Horse Races (at track or off-track)*	NR	17.0	19.8^b^	35.7	NR
Games of Skill (e.g., pool, darts, etc.)	17.1	22.3	23.5	27.9	9.2^b^
Dare or Challenge*	14.0	24.6	25.0	25.0	11.5
Bingo	16.3^b^	22.8	23.0^b^	22.7	15.3^b^
Internet Gambling Behaviours Played *for Fun (no money)*					
Internet Poker	16.1	20.0	21.8	30.1	12.0
Internet Slots	18.6	22.4	22.1	27.9	9.0^b^
Gambling Games on Facebook	18.3	26.1	21.2	25.9	8.5^b^

^a^Weighted population estimate

^b^Moderate sampling variability, interpret with caution

^NR^High sampling variability or low sample size, data are suppressed

* = *p* ≤0.05, ** = *p* ≤0.01, *** = *p* ≤0.001

### Frequency of gambling

As shown in Table 3, in every gambling activity a higher proportion of adolescents gambled at least monthly but less than weekly suggesting that adolescents are not gambling frequently.

**Table 3:** Prevalence of participation in gambling behaviours by frequency, Grades 9 to 12 students in the Youth Gambling Survey (YGS) Saskatchewan, Ontario, and Newfoundland and Labrador (Canada, 2012-2013)

	*Current Participation (past 3 months)*		
	At Least Weekly	At Least Monthly	Total
Gambling Behaviours Played *for Money*	%^a^	%^a^	%^a^
Internet Gambling			
Internet Poker	1.8	1.9	3.7
Sports Pools (e.g., Hockey, Baseball) Online	2.5	4.8^b^	7.3
Slot Machines Online	1.2	1.2	2.4
Land Based Gambling			
Sports Pools (e.g., Hockey, Baseball) Not Online	2.7	6.2	8.9
Slot Machines Not Online	1.1^b^	NR	1.9
Arcade or Video Games	2.3	7.3	9.6
Sport Select (e.g., Pro Line, Over/Under)	2.4	4.7^b^	7.1
Lottery Tickets (e.g., 6/49, Super 7)	2.0^b^	7.6	9.6
Instant-Win or Scratch Tickets	2.1	11.6	13.7
Cards (e.g., Poker, Black Jack)	1.9	6.8	8.7
Board Games or Dice	2.0	3.8	5.8
Video Lottery Terminals	1.1^b^	1.3^b^	2.4
Horse Races (at track or off-track)	1.2^b^	1.8	3.0
Games of Skill (e.g., pool, darts, etc.)	3.1	9.4	12.5
Dare or Challenge	3.6	18.5	22.1
Bingo	1.7	5.1	6.8
Internet Gambling Behaviours Played *for Fun (no money)*			
Internet Poker	3.0	6.1	9.1
Internet Slots	1.7	3.3	5.0
Gambling Games on Facebook	3.0	6.0	9.0

^a^Weighted population estimate

^b^Moderate sampling variability, interpret with caution

^NR^High sampling variability or low sample size, data are suppressed

### Free simulated gambling prevalence

Adolescents also participated in free simulated gambling activities; 9.1 % reported playing internet poker for no money, 4.9 % reported playing internet slots for no money, and 9.0 % reported playing gambling games on Facebook. Again, males were significantly more likely to report engaging in each of these activities compared to females. Table 4 reports the proportion of adolescents engaging in simulated gambling games by gambling status. Among those who did not report engaging in any gambling for money, 5 % had played internet poker for free, 2.3 % had played internet slots,^1^ and 4.8 %^2^ had played gambling games on Facebook. A significantly higher proportion of those playing gambling games for money had also reported playing free simulated gambling games: 14.7 % of current gamblers had played free internet poker, 8.6 % had played free internet slots, and 14.6 % had played gambling games on Facebook.

**Table 4:** Prevalence of simulated gambling by gambling status, Grades 9 to 12 students in the Youth Gambling Survey (YGS) Saskatchewan, Ontario, and Newfoundland and Labrador (Canada, 2012-2013)

	*Current Participation (past 3 monthsd*		
	Non-Gamblers	Gamblers	
	(*n* = 5902)	(*n* = 3928)	Chi-Square
Internet Gambling Behaviours Played *for Fun (no money)*	%^a^	%^a^	
Internet Poker	5.0	14.7	*X* ^*2*^ = 52.3(1), *p* < 0.001
Internet Slots	2.3^a^	8.6	*X* ^*2*^ = 51.2(1), *p* < 0.001
Gambling games on Facebook	4.8^a^	14.6	*X* ^*2*^ = 38.1(1), *p* < 0.001

^a^Moderate sampling variability, interpret with caution

### Online gambling among adolescents who had participated in gambling

**Fig. 1:**
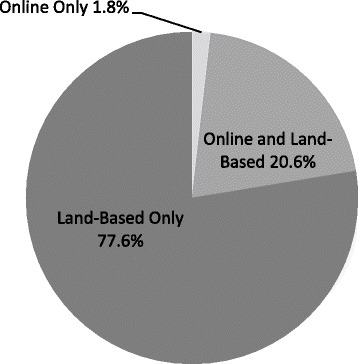
Online and Land-Based Gambling Prevalence, Youth Gambling Survey Saskatchewan, Ontario, and Newfoundland and Labrador (Canada) 2012-2013

Among adolescents who had participated in gambling, 22.4 % had gambled money or something of value online. Males who had gambled (31.8 %) were significantly more likely to gamble online compared to females who had gambled (10.3 %) (*χ*^2^ = 24.5, *df* = 1, *p* < 0.001). As reported in Fig. 1, few gamblers gamble online exclusively (1.8 %) but 20.6 % of those who had gambled participated in both online and land-based gambling. The majority of adolescent gamblers participate in land-based gambling only (77.6 %).

**Fig. 2:**
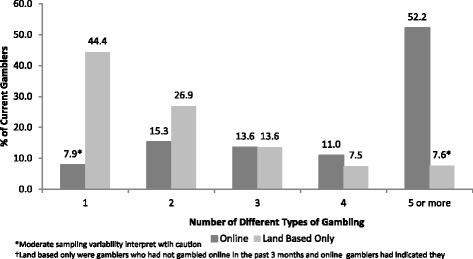
Number of Types of Gambling by Online (*n*=833) vs. Land Based Gamblers(*n*=3041)† Youth Gambling Survey Saskatchewan, Ontario, and Newfoundland and Labrador (Canada) 2012-2013

Figure 2 reports the number of different types of gambling games played among online vs. land-based gamblers. Online gamblers were significantly more likely to participate in multiple gambling modes compared to land-based gamblers (*χ*^2^ = 420.4, *df* = 4, *p* < 0.001). Of the 833 youth who reported gambling online, the majority (52.2 %) reported that they participated in 5 or more different types of gambling activities compared to 7.6 %^3^ of the 3,095 land-based only gamblers.

**Fig. 3:**
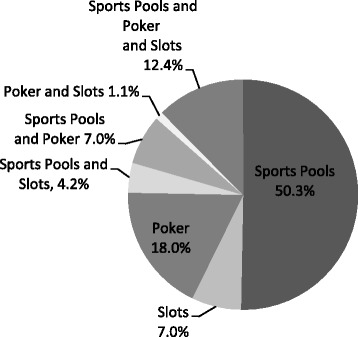
Online Gambling Participation by Type (n=833), Youth Gambling Survey Saskatchewan, Ontario, and Newfoundland and Labrador (Canada) 2012-2013

Figure 3 reports online gambling participation by game type. The majority of online gamblers gambled exclusively on sports pools online (50.3 %), whereas 12.4 % of adolescent internet gamblers had gambled on all three forms of online gambling assessed: internet poker, online slots and sports pools.

### Problem gambling prevalence by online vs. land based gambling

Table 5 reports problem gambling prevalence by online compared to land based gambling participation. A significantly higher proportion of adolescents who indicated that they participated in online gambling in the past 3 months were classified as “high” or “low to moderate risk” for problem gambling compared to adolescents who exclusively participated in land-based gambling (*X*^2^ = 128.07 *p* < 0.001). Among online gamblers, 17.4 % scored “high” and 18.2 % scored “low to moderate” in gambling severity whereas 1.2 %^4^ of land-based only gamblers scored “high” and 7.2 % scored “low to moderate” in gambling severity.

**Table 5:** Problem Gambling by Online vs. Land Based Gambling^a^ Grades 9 to 12 students in the Youth Gambling Survey (YGS) Saskatchewan, Ontario, and Newfoundland and Labrador (Canada, 2012-2013)

Problem Gambling Severity Subscore (CAGI)	Online Gambler (*n* = 758)	Land-Based Only Gambler (*n* = 2924)	Chi-Square
No problem gambling	64.4 %	91.6 %	*X* ^*2*^ = 128.07 *p* < 0.001
Low to moderate problem gambling	18.2 %	7.2 %	
High gambling severity	17.4 %	1.2%^b^	

^a^Land based only were gamblers who had not gambled online in the past 3 months and online gamblers had indicated they gambled online in the past 3 months

^b^Moderate sampling variability interpret with caution

## Discussion

Among adolescents in Grades 9 to 12 in Ontario, Saskatchewan and Newfoundland and Labrador, 41.6 % had gambled and 9.4 % had gambled online in the past 3 months alone. Consistent with research in other jurisdictions, online and land-based gambling was significantly more popular among males than females [13, 16, 21]. Results were not significantly different by province. Despite restrictions on online gambling, based on the current data an estimated 58,000 adolescents have gambled online. It is also worth noting that at the time of the survey online gambling had not been legalized in any of the provinces although each province did allow sports betting such as “Pro-line” and “Sports Select” on each of the provincial websites. Sports pools online were the most common form of online gambling reported. It is unknown, however, which websites youth were using to access online sports pools or whether this included activities such as fantasy sports pools conducted online. Future research examining how adolescents are engaging in online sports pools is needed. However, this data can be used as a baseline to compare to subsequent studies to see how legalization of online gambling for adults over 19 years of age will affect youth gambling.

The prevalence of online gambling is significantly higher than has previously been found despite the fact that we used a shorter time frame (past 3 months vs. past 12 months used in the OSDUHS) [16]. A chi-square test of the difference in prevalence (9.4 % vs. 3.0 %) was 219.26 and therefore is significantly different (*p* < 0.01). One potential explanation for these differences could be how we assessed online gambling. Whereas previous surveys have asked respondents to report about their online gambling overall, we asked respondents about 3 specific types of online gambling: internet poker, sports pools online and slot machines online. It is possible that when respondents are asked to consider their online gambling they don’t immediately think of sports pools online as a form of online gambling and therefore under-report their online gambling behaviour.

Online gambling is associated with problem gambling among adolescents. Compared to adolescents who exclusively participated in land-based gambling, a significantly higher proportion of online gamblers were classified as “high” or “low to moderate in problem gambling severity”. This is noteworthy particularly given that this is the first study to use a measure of problem gambling developed specifically for adolescents. Few adolescents participated in online gambling exclusively and online gamblers were more likely than land-based gamblers to participate in multiple forms of gambling. These findings are consistent with the hypothesis that online gambling could be problematic for those who have a problem with gambling more generally [22].

Adolescents are also using online platforms to engage in simulated gambling. Internet poker and gambling games on Facebook were the most popular forms of free online gambling. This is a cause for concern because many online gambling websites use these free demo games to recruit new gamblers to pay sites. Additionally, Facebook has indicated that they may integrate online gambling into Facebook if the United States legalizes online gambling and they have already done so in the United Kingdom [23]. Thus, by the time Facebook provides online gambling to youth in Canada, many will have already participated in free games and will already be comfortable with using it. Free games would also be expected to normalize gambling given their widespread use. The findings from this study indicate that a higher proportion of adolescents who were playing free online games were also gambling for money. This may be indicative of an overall pattern of problematic gambling.

Gambling on the outcome of video games is also one of the most popular forms of gambling for males (14.5 %). Further research is needed to understand whether adolescents are betting on the outcomes of video games or engaging in video games that include gambling for money, or both. What is known, is that the lines between gambling games and video games have become blurred as technology has changed [24]. Researchers have therefore become concerned that engaging in gambling with video games may increase the likelihood that gambling is viewed as more socially acceptable, may increase positive attitudes towards gambling, and could potentially increase the likelihood of problem gambling in the future [24]. The research on online gambling is still in an early stage in a rapidly changing commercial area. Given the increased legalization of online gambling, this topic needs to be explored further to test whether or not these concerns about youth access to the internet for gambling are substantiated. However, the current study demonstrates that given the popularity of video games for gambling particularly among adolescent males, further research is urgently needed.

Consistent with previous research [25] most youth still gamble mostly on non-commercial forms of gambling such as dares or challenges. There was some variability in gambling participation by age. It is worth noting, however, that the highest proportion of adolescents participating in many of the forms of gambling were not of legal age. The exception was lottery tickets and instant-win/scratch tickets. However, it is important to note that the majority of adolescents who had gambled with lottery and scratch tickets were not of legal age to purchase them. Although previous research has consistently identified lottery and scratch tickets as popular forms of gambling for youth [13], lottery corporations in each of the provinces have indicated a concern that ticket-based gambling was no longer appealing to the “video game generation” [26]. The Interprovincial Lottery Corporation is therefore looking for new ideas for a “new national lottery game that will be attractive to the 18-34 year old player base” [26]. Given that our study demonstrates that these games are already popular it is therefore important to ensure that these strategies do not encourage further gambling engagement among underage youth.

A higher proportion of adolescents gambled infrequently (at least monthly but less than weekly). An argument could be made that infrequent betting by youth is less of a concern than if adolescents were gambling more frequently. However, research has demonstrated that early gambling initiation (before the age of 21) is associated with more problematic gambling in the future [27]. Therefore, it is important to examine both overall participation in gambling and the frequency of gambling among adolescents.

The government of Ontario launched their own legalized online gambling platform in January 2015. Although many jurisdictions have already launched government-run online gambling programs and most provinces in Canada have publicly expressed an interest in legalizing online gambling, the impact of government-run online gambling is unknown. Governments argue that legalization would allow online gambling to be regulated, that vulnerable populations such as adolescents would be more protected and that online gambling would generate tax revenues [28]. Indeed, previous studies have suggested that few safeguards exist for protecting underage youth from gambling on many online gambling websites and that the major barrier to online gambling is payment [29]. However, it is possible to gamble online using a PayPal account, wire transfers, single use credit cards, etc. Efforts to restrict youth gambling by ensuring age checks such as those used by the PlayOLG website may be more effective at restricting access. It is also possible that youth are accessing more unregulated forms of online gambling such as fantasy sports with friends online. Further research is needed to identify which websites youth are accessing.

However, legalization could also provide legitimacy to online gambling and prevalence rates could increase. The simple prevalence data presented here provide important baseline levels of gambling behaviour among Ontario youth prior to the legalization of online gambling. The results indicate that rates of online gambling are higher than expected based on previous estimates and considering the fact that our measure examined online gambling in the past 3 months alone. Regulations to limit online gambling participation among adolescents therefore need to be strengthened.

### Limitations

This study has several limitations common to survey research. Although the response rate was high and the data were weighted to help account for non-response, the findings are nevertheless subject to sample bias. In addition, the findings may reflect some underreporting for gambling behaviour common in survey research. However, research suggests that impression management effects may be an issue for self-reported problem gambling but not gambling behavior [30]. YGS data are also based on self-reported measures taken from CAGI, which have previously demonstrated satisfactory reliability and validity [20]. Honest reporting was also encouraged by ensuring confidentiality during data collection. It should also be noted that the cross-sectional nature of the design does not allow for causal inferences regarding trends over time. Longitudinal data are required to determine the temporal sequence of the onset of these gambling behaviours.

## Conclusions

This study clearly shows that many youth are gambling online despite restrictions. Further restrictions and harm reduction approaches are needed to ensure that the wide availability of online and simulated gambling does not lead to increases in problem gambling. Longitudinal research is also needed to explore whether gambling online and use of simulated online gambling leads to subsequent gambling behaviour and problem gambling or whether problematic gambling leads to participation in both gambling for free and for money. Results from this research suggest that it is likely that problem gamblers are participating in multiple forms of gambling, including online gambling.

## Footnotes

1 Moderate sampling variability, interpret with caution.

2 Moderate sampling variability, interpret with caution.

3 Moderate sampling variability, interpret with caution.

4 Moderate sampling variability, interpret with caution.
